# Draft Genome Sequence of the Oleaginous Yeast *Yarrowia lipolytica* PO1f, a Commonly Used Metabolic Engineering Host

**DOI:** 10.1128/genomeA.00652-14

**Published:** 2014-07-03

**Authors:** Leqian Liu, Hal S. Alper

**Affiliations:** a Department of Chemical Engineering, the University of Texas at Austin, Austin, Texas, USA; b Institute for Cellular and Molecular Biology, the University of Texas at Austin, Austin, Texas, USA

## Abstract

The draft genome sequence of the oleaginous yeast *Yarrowia lipolytica* stain PO1f, a commonly used metabolic engineering host, is presented here. The approximately 20.3-Mb genome sequence of PO1f will greatly facilitate research efforts in metabolic engineering of *Yarrowia lipolytica* for value-added chemical production.

## GENOME ANNOUNCEMENT

*Yarrowia lipolytica*, a nonconventional oleaginous yeast, has recently emerged as a potential host strain that is recognized both as safe (1) and as a potent producer of value-added chemicals and industrial protein (2, 3). As a result, there has been a growing interest in biotechnological applications in this host strain due to both established biological information and intriguing physiological characteristics. Recent efforts have further expanded the genetic toolbox for *Y. lipolytica* (4, 5) and rewired metabolic networks for high-level production of fatty acid-based value-added chemicals (6–9).

Although a high-quality genome sequence of *Y. lipolytica* strain CLIB122 (E150) has been available (10), this strain is not the most popular for metabolic engineering applications. Specifically, *Y. lipolytica* strain W29 (CLIB89) and its derivatives, such as PO1f, have been more widely used, especially in metabolic engineering studies for value-added chemical production (6, 7, 9, 11–13), therapeutic protein production (14, 15), and fundamental microbiology studies (16–18). As one of the parental strains of the French inbred lines, the wild-type haploid strain, W29, was originally isolated from sewage material (19). A preliminary sequencing effort was conducted with only 4.9 Mb available (20). To gain a better understanding of the strain W29 and its potential for value-added chemical production, we generated the genome sequence for its derivative strain, PO1f.

The genome of *Y. lipolytica* PO1f was sequenced using the Illumina HiSeq DNA sequencing platform (PE2X100). The raw sequence data comprise a total of 8,740,022 reads that together provide very high sampling coverage of the genome (43.7-fold coverage). The reads were assembled using Velvet with a k-mer size of 55 (21). This led to a genome assembly containing 669 contigs (each at a length of ≥500 bp). The total length of the genome assembly is 20,282,994 bp, with an *N*_50_ equal to 58 kbp. The reads were also assembled using the A5 pipeline (22), and gaps were closed with IMAGE (23) to 348 contigs (each at a length of ≥500 bp) and further scaffolded based on the genome sequence of strain CLIB122 using ABACAS (24). A total of 19,922,824 bp was placed to the final 6 scaffolds.

The final *de novo* assembled genome was analyzed to assign open reading frames (ORFs) with Augustus (25) trained with *Y. lipolytica* CLIB122 data. A total of 6,420 putative ORFs were identified and 4,096 were annotated with Blast2Go (26). The genome sequences of PO1f and strain CLIB122 are very similar in nature. By mapping the Illumina reads to the CLIB122 genome using BWA (27) and analyzing using Samtools (27) and BEDTools (28), a total of 24,675 single nucleotide variations were called in PO1f genome sequences (QUAL ≥30; DP ≥10). Long terminal repeat (LTR)-retrotransposon elements are confirmed to be absent in strain PO1f, matching prior information about this strain (20). There is one large deletion in chromosome A with four ORFs missing. Two of them are weakly similar to the SMC5/6 complex (YALI0A01562p and YALI0A01602p), which are related to double-strand break repairing and homologous recombination (29). These absences may give rise to differences in homologous recombination efficiencies in this strain.

### Nucleotide sequence accession numbers.

This whole-genome shotgun analysis has been deposited at DDBJ/EMBL/GenBank under the accession no. JAFI00000000. The versions described in this paper are versions JAFI01000000 and JAFI02000000.
